# Royal Chest Hospital, City Road

**Published:** 1902-03-22

**Authors:** 


### ROYAL CHEST HOSPITAL, CITY ROAD.

### NURSES' NEW HOME.

The Royal Hospital for Diseases of the Chest, City Road, wore quite a festive air on Friday, 14th inst. There were pots of flowers in the entrance, and in the windows overlooking the main thoroughfare, while inside, in the Board Room, the matron had evidently been at work, beautifying table, windows, and chimney-piece with daffodils and genesta in ovals and pots.

At three o'clock the Lord Mayor and the Lady Mayoress, and Mr. Alderman Sheriff Bell, arrived, and were shown round the wards, with which they were evidently much pleased, for the Lord Mayor gave a special donation of £21, and Mr. Alderman Bell gave five guineas as an annual subscription, and a donation of the same amount. The governors, some of whom accompanied the Lord Mayor through the wards, were present to the number of about eighteen, three being clergymen and three ladies.

It was not much before the half-hour struck that the business began, and the Lord Mayor moved the adoption of the report and accounts.

### The Nurses' Home.

For some time past the council of King Edward's Fund, and the medical staff of the hospital itself, have been urging the Committee of Management to provide a home, apart from the hospital, for the sisters and nurses, who at present sleep immediately over the wards. The sum required for building and furnishing, on the site successfully negotiated for by Mr. George Sandeman and his partners (Honorary Solicitors), is £7,000, and a special appeal has been issued for the purpose by the treasurer, Mr. Hope Morley.

"There is one matter," said the Lord Mayor, "which I know is occupying the attention of the governors of this hospital, and that is the absolute necessity of a nurses' home. The House Committee have been fortunate enough to acquire a site. It remains for the benevolent public to find the wherewithal for the building. I suppose anyone who has the smallest smattering of knowledge concerning the disease treated here knows that it is absolutely necessary for the nurses to sleep in pure air, quite away from the hospital itself."

The Lord Mayor then appealed, as his custom is, through his "old friends the Press" for funds to build and furnish a nurses' home on the site already acquired. This, he said, would bring the hospital up to date, and was necessary for any hospital if it was to be a success. He had been connected with the London hospitals for many years, and had often taken the chair at festival dinners. It was very hard work getting up the subscriptions, and he thought he had not done badly at it. But never, in the whole course of his experience, had he raised such a magnificent sum as that obtained by Sir Andros de la Rue at the Hôtel Métropole, last May, for the purpose of the nurses' home. The receipts amounted to £5,858 3s. 9d. While on the subject of finance he would like to call attention to the loss of £85 in annual subscriptions, because annual subscriptions were the life-blood of an institution. It was the guineas and half-guineas that mounted up and made the hospital's reliable income.

### Beds for Out-Patients.

The motion was seconded by Mr. Forde Ridley, M.P. for the S.W. division of Bethnal Green, who said he was justly proud of the sum collected by the working men of his constituency, namely, £168, an assurance, he thought, that the poor really appreciated a hospital conducted on the lines of the Royal Chest Hospital, for there was no class more critical of the way their money was spent. There was one point which appeared to him of especial importance, viz., that a certain number of beds were kept for serious out-patient cases requiring immediate admission. There was no work more noble and splendid, and he could speak from personal knowledge of the benefit derived by young men in whom the symptoms of the disease had developed so rapidly as to make immediate admission an absolute necessity. At other hospitals there might be a delay of weeks, if not months, but here, if the case was urgent, an out-patient's letter sufficed for immediate admission, and he believed that if the proper treatment were applied at once it was possible to effect a complete cure.

The ordinary business of reappointments and votes of thanks to honorary officers having been transacted, the court adjourned. A feature at this hospital is the reading, after the notice convening the meeting, of the "Laws relating to business to be done at Annual Courts."

